# New Appliances and Things Medical

**Published:** 1909-05-22

**Authors:** 


### NEW APPLIANCES AND THINGS MEDICAL.

[We shall be glad to receive at our Office, 28 & 29 Southampton Street, Strand, London, W.C., from the manufacturers, specimens of all new preparations and appliances.]

### "VAPOROLE" AROMATIC AMMONIA.

Messrs. Burroughs and Wellcome have sent us samples a convenient means of instantly obtaining ammonia for halation in cases of fainting, etc. "Vaporole" Aromatic monia presents a solution of ammonia gas with aromatics dy for immediate use in emergency. This ingenious le appliance should prove more reliable than ordinary nelling salts, which rapidly deteriorate. Each "Vapo- le" Aromatic Ammonia contains a small quantity of solu- 1 in a capsule surrounded by absorbent material, the ole being enclosed in a covering of silk. When required little capsule, which resembles that used for Amyl rite, may be fractured by pressure between the fingers the vapour inhaled. "Vaporole" Aromatic Ammonia ssued in aluminised boxes of 12, which easily fit the istcoat pocket. Medical men and dental surgeons will doubt welcome this novel and portable form of smelling ts. The vapour is pleasant and effective.

### FRY'S COCOA AND CHOCOLATE.

Messrs. J. S. Fry and Son, Ltd., of Bristol, have sent us lately a sample of their excellent Pure Concentrated Cocoa, which has long been recommended by the medical profession for its solubility, pleasant taste, and digestibility. They also forwarded a box of a new variety of sweetmeat, which they have supplied to the King and Queen of Spain, and have named "Reina Victoria Eugénie" Chocolates. These are mixed chocolates—very dainty in appearance and excellent in flavour, and packed in artistic boxes.

Mr. Joseph Chapman, of Cleethorpes, who died last week, leaving estate approximately worth £250,000, bequeathed large legacies to the Brompton Hospital and other London hospitals, while the residue of his estate reverts to the Grimsby and District Hospital, and will amount to several thousands of pounds.

